# In-line monitoring of surfactant clearance in viral vaccine downstream processing

**DOI:** 10.1016/j.csbj.2021.03.030

**Published:** 2021-03-26

**Authors:** Jessie Payne, James Cronin, Manjit Haer, Jason Krouse, William Prosperi, Katherine Drolet-Vives, Matthew Lieve, Michael Soika, Matthew Balmer, Marina Kirkitadze

**Affiliations:** a Analytical Sciences, Sanofi Pasteur, Toronto, Canada; b Queen's University, Kingston, Canada; c Mettler Toledo AutoChem Inc., Columbia, USA; d Manufacturing Technology, Sanofi Pasteur, Swiftwater, USA; e Viral Manufacturing, Industrial Affairs, Sanofi Pasteur, Swiftwater, USA; f Analytical Sciences, Sanofi Pasteur, Swiftwater, USA

**Keywords:** Viral vaccine, Residuals, IR, ReactIR, PAT, Clearance

## Abstract

**Purpose:**

The goal of this study is to examine the suitability of in-line infrared measurements to monitor, in real-time, surfactant concentration in the viral vaccine drug substance during a 50KDa tangential flow filtration (TFF) process.

**Methods:**

A ReactIR™ 702L instrument was used to gather spectra of process off-line samples and reference materials to assess the feasibility of monitoring surfactant concentration during a TFF process in real-time. Both univariate and multivariate models were used to evaluate the off-line sample data and were found to be in good agreement with surfactant concentration values obtained by HPLC. These results were used as justification for a real-time TFF experiment with live process material.

**Results:**

Small scale ReactIR experiments with process material demonstrated that a multivariate model using the 1300 cm^−1^ to 1000 cm^−1^ spectral region can be used to predict surfactant concentrations between TFF exchanges 8 to 15.

**Conclusion:**

The results of this study demonstrated suitability of an in-line infrared measurement to monitor surfactant concentration in the viral vaccine drug substance between exchanges 8–15 of a 50 kDa tangential flow filtration process. The preliminary multivariate model used for this work can be further optimized for the in-line use at manufacturing scale.

## Graphical abstract

**Figure:**
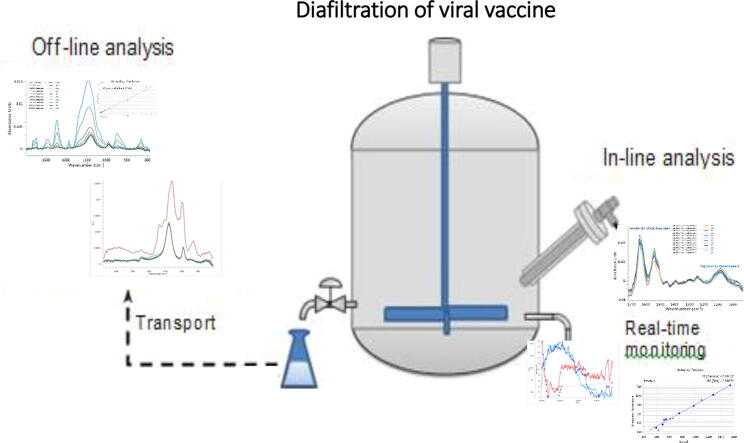

## 1 Introduction

**Fig. 1:**
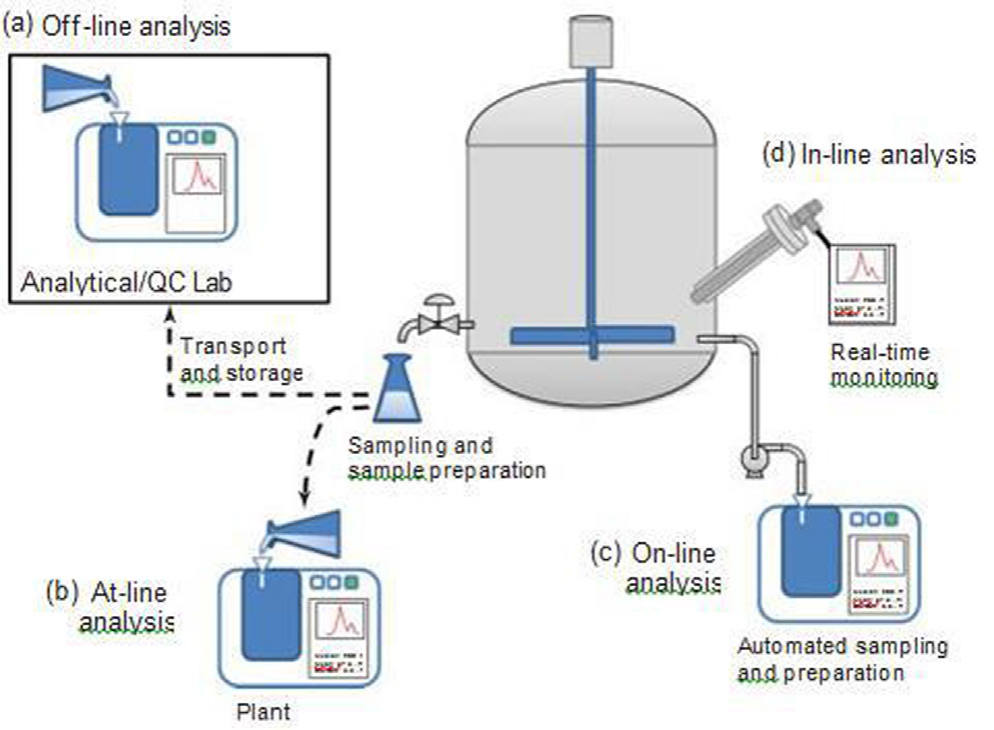
Schematic diagram demonstrating analysis using PAT. Courtesy of Mettler Toledo.

Process analytical technology (PAT) has been defined by the United States Food and Drug Administration (FDA) as a mechanism to design, analyze, and control pharmaceutical manufacturing processes through the measurement of Critical Process Parameters (CPP) with Critical Quality Attributes (CQA) of the product [1]. PAT instruments include on- and in-line analyzers capable of measuring physical and chemical process parameters and critical material attributes with the goal of optimizing process control. In the form of a probe, PAT is routinely designed into the manufacturing line by insertion into the tanks and vessels (Fig. 1). These in-line measurements collect data directly from the processes occurring in these areas. In some cases, the probe or sensor cannot be inserted directly into a manufacturing vessel due to sterility constraints, or simply facility design constraints. In these cases, PAT can be implemented on-line; where a small amount of material is diverted, while maintaining sterility, from the main manufacturing line into an offshoot vessel and analyzed alongside where a critical process is taking place (Fig. 1). PAT is often coupled with computational methods to perform multivariate modelling for both spectroscopic results and multilayer statistical control of the processes.

The implementation of process analytical measurements is routinely justified by reduction of the manufacturing cycle time due the use of on- and in-line measurements and controls, reduction or elimination of failed batches, improved control of the process and product quality in real-time. Current in-process tests employ laboratory equipment that require time consuming, and expensive operation, method validation, manual sampling, large quantities of testing material, and maintenance. Unlike laboratory tests, PAT can produce rapid results which can reduce or eliminate the need for manual sampling. This provides manufacturing scientists with the opportunity to make real-time decisions to optimize quality and throughput in product manufacturing [2] at various manufacturing stages; including, fermentation [3], [4] and purification. Additionally, the implementation of a PAT solution minimizes potential safety hazards associated with manual sampling and the current requirement of direct analyst involvement.

Quality control (QC) laboratory release testing, however, is a critical and mandatory aspect of vaccine manufacturing. Independent QC testing is in place to ensure test articles are produced consistently within batch manufacturing specifications and that products are efficacious and safe for patients. The Code of Federal Regulations [5] requires these tests, such as potency, microbial safety, and content, to occur outside of the manufacturing department to ensure independent batch release. The assessment of test articles, raw materials, and reference standards are strictly controlled, consume considerable testing volume, and require rigorous validation, robustness and trending. As a result, there is often a conflict when manufacturing scientists propose real-time data collection outside of the specification tests that are the sole responsibility and focus of QC, as it further complicates the process of assessment. The implementation of PAT circumvents this obstacle by allowing scientists to trend large data sets to visualize the effect of process change and to facilitate continuous improvement through reduction of lost product and increase of plant uptime; without interfering with current QC testing.

PAT spectroscopic analyzers, such as in-line near and mid-infrared (MIR) spectroscopy and Raman spectroscopy produce large data sets reflecting process conditions in real time; however, this information is not readily interpretable as is. Understanding this multilayer process information becomes integral in utilizing the knowledge gained from the implemented PAT and initiating the decision feedback loop in minimal time. Key to this understanding is the application of chemometric analyses to process large data sets and determine relationships between product and process parameters [6], [7]. These analysis methods include multivariate curve resolution [8], partial least square regression (PLS), and more complex multivariate statistical process control (MSPC) [9], [10]. These methods can be used to combine large, dissimilar data sets to predict process critical quality parameters and alert an analyst to out of specification results.

The FDA’s release of the PAT guidance document [1] stimulated the development of novel PAT applications for improved process monitoring and understanding. Early biologics PAT literature reports were centered on improvements in CHO cell culture productivity [3]. Raman spectroscopy was prominent in these early publications and has been successfully used to model metabolites in CHO cell culture monitoring [3]. NIR has also been successful in modelling [4] metabolites in CHO cell culture studies. Variables that influence bacterial and viral growth, process metabolites, and yield such as concentration, pH, and gas flow, can also be measured in real-time with spectroscopic methods [11]. Chemometric modeling can then be used to predict production yield based on the data collected by PAT, such as NIR and Raman [11].

The broad success of in-situ spectroscopic measurements in upstream applications has led to investigations into improving process understanding and control in common downstream unit operations. Unlike long duration upstream measurements that can range from days to weeks, downstream processing steps are normally less than a day and can be as short as few hours. As a result, requirements for downstream measurements are substantially different with an emphasis on speed of response, ease of use and utility for more than one operation. Downstream processing involves the purification and recovery of product and includes the removal of insoluble materials, product isolation, product purification, residual clearance, and final product formulation. The clearance and reduction of process residuals is a common, vital step in downstream processing. These residuals must be monitored closely as they can affect product efficacy or cause adverse reactions in patients, and the stringent regulations surrounding their clearance and reduction reflect this.

Consideration of appropriate measurement options to evaluate included Raman and Infrared spectroscopy. While both Infrared and Raman spectra of surfactant exhibit spectral differences compared to the sample matrix, the longer measurement time (8 to 12 min) required by Raman spectroscopy was found to be only a minimal improvement compared with the incumbent HPLC method. In addition, Raman spectra were susceptible to a changing fluorescence background from the sample matrix. Compared with other techniques such as NIR and Raman, the lack of interference from suspended solids (scattering) and the fixed pathlength of ATR probes tends to reduce the number of variables required in multivariate IR models For these reasons, a further evaluation of infrared spectroscopy was initiated with a goal of providing a real-time in process measurement of surfactant concentration. If the components of interest have unique IR absorbances, univariate Beer's law-based models are simple to implement and are routinely accurate enough to provide concentration vs time trends for major components of a process. For processes in which infrared spectra of the components of interest may be complex and contain overlapping peaks, the use of multivariate models such as partial least squares (PLS) are common. Creation of multivariate models requires careful selection of training set standards that represent concentration ranges of each component and the elimination of covariance between components of multicomponent systems.

**Fig. 2:**
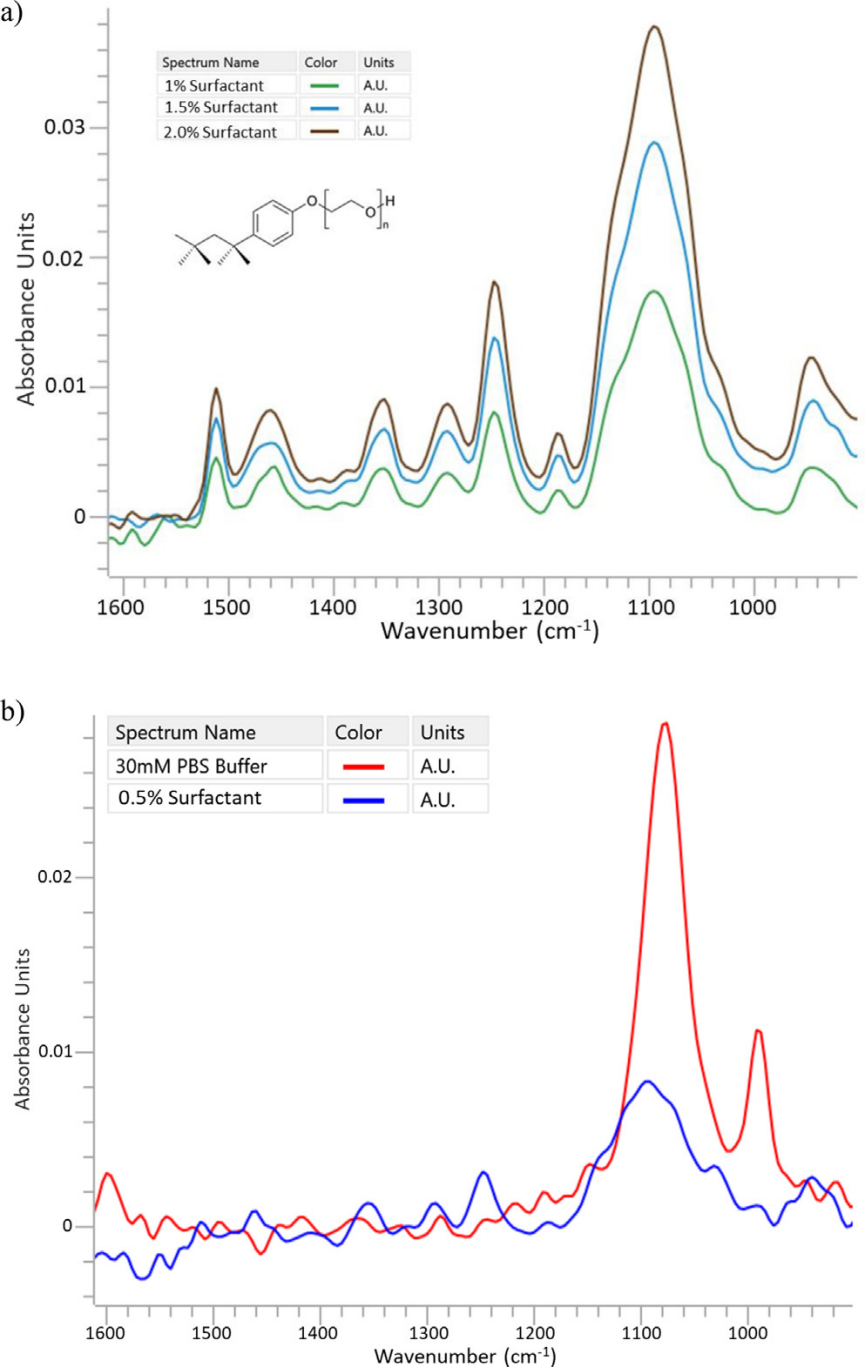
a) Surfactant Standards in water. FTIR spectra of 2% Surfactant (dark red trace), 1.5% Surfactant (blue trace), and 1%. b) Reference spectra of 30 mM PBS buffer (red trace) and surfactant (blue trace) with water subtracted 0.5% Surfactant (blue trace). (For interpretation of the references to colour in this figure legend, the reader is referred to the web version of this article.)

The scope of this study includes a critical downstream buffer exchange during a tangential flow filtration (TFF) process in the production of viral vaccine. In a prior process step, a non-ionic surfactant, 2-[4-(2,4,4-trimethylpentan-2-yl)phenoxy]ethanol, hereby referred to as surfactant (see Fig. 2), is added to facilitate separation of host proteins from the viral product. In this step, the initial buffer is exchanged for PBS and the surfactant concentration is reduced from approximately 4 g/L to 1 g/L. The TFF process typically exchanges fifteen diavolumes (15 min/diavolume) across a 50 kDa membrane to concentrate the retentate to its target and to reduce the surfactant to the desired concentration. During the first 6 to 7 exchanges, the starting buffer which has a high sucrose concentration is exchanged for a PBS buffer. Surfactant does not pass as efficiently through the membrane as sucrose. The diafiltration of detergents is typically difficult due to interactions with other detergent molecules in micelles and due to interactions with hydrophobic groups on the product. As such, the decrease of surfactant concentration become noticeable after the 8th cycle, that is from diavolume 8 to diavolume 15. During the first half of the process the sucrose was completely removed, and the surfactant concentration is reduced from approximately 4 g/L to 2 g/L. The remaining exchanges, 8 through 15 are normally required to reduce the surfactant concentration to the target of approximately 1 g/L.

At present, HPLC analysis is used to determine concentration of surfactant in the in-process diafiltration step of viral drug substance. At scale, the TFF process requires approximately 15 min per volume exchange. Currently, an off-line sample is collected at the end of each diavolume and analyzed using a rapid HPLC method to determine concentration of surfactant in each of the diavolume off-line samples. A rapid, accurate measurement of the relatively low concentrations of surfactant in a matrix of host proteins, virus proteins and changing buffer compositions is a technical challenge. The current chromatographic measurement provides the required specificity and accuracy but requires collecting a representative sample and the response times can vary from 20 to 30 min excluding complications. Further, a trained operator must be available to calibrate, maintain and operate the HPLC at any time a TFF process is running. Therefore, the goal of this study was to develop an in-line IR measurement that approaches the precision and accuracy of the incumbent HPLC method, which would be of approximately ± 0.1 g/L of surfactant measurement.

## 2 Materials and methods

### 2.1 Materials used in the study

The aliquots of drug substance lots used in this study were produced in-house at manufacturing scale. In the drug product, which is not in scope of this study the concentration of surfactant is significantly lower after approximately 20-fold dilution and is measured by HPLC.

### 2.2 Lab FTIR spectrometer

Off-line IR spectra were recorded using a Nicolet iS50 FTIR (Thermo Fisher Inc., USA) equipped with an ATR module. No further sample preparation was required and approximately 12 µl of sample was transferred directly onto the diamond crystal for analysis. A spectrum was collected for each sample, cleaning the crystal in between the sample measurements. Data acquisition was performed using OMNIC software (Thermo Fisher Inc., USA) and was plotted in absorbance units. This data was then analyzed using TQ Analyst (Thermo Fisher Inc., USA).

### 2.3 Standards and process off-line sample information

Surfactant standards were created at concentration ranging from 1% to 2% to identify peak locations. Infrared spectra of the surfactant standards and a series of process samples from 4 different TFF runs, were collected on a Mettler Toledo 702L ReactIR™ with a 9.5 mm DiComp probe with a 1.5-meter fiber at 8 cm^−1^ resolution and 256 scans. The spectral data were plotted in absorbance units and displayed with water subtracted and or PBS buffer subtracted. The intermediate precision of the off-line IR method is below 2%.

### 2.4 ReactIR small scale TFF experiment

IR spectra were recorded using a ReactIR 702L (Mettler Toledo Inc., USA) equipped with a 9. 5 mm diameter DiComp ATR probe with a 1.5-meter fiber. Spectra were collected at 8 cm^−1^ resolution and 512 scans (3 min). The probe was submerged in the retentate vessel and data collected throughout the TFF run. A reference spectrum of the final PBS buffer was collected and used to subtract from the TFF experiment data. With no initial concentration, a 14X (equal volume) diafiltration was performed using 30 mM PBS buffer at RT. 1 mL retentate samples were pulled throughout the TFF process and tested via HPLC. There was no TFF rinse post diafiltration. After the 14th exchange, 0.5 mL of 10% surfactant solution was spiked back into the post 50 kDa material a total of 5 times. Additions 1–4 were mixed for 15 min and addition 5 mixed overnight. IR readings and HPLC samples were taken after each mix.

## 3 Results

### 3.1 Surfactant standard

The spectra of the surfactant standards were found to be consistent with the structure of the molecule. The most intense, broad absorbance near 1100 cm^−1^ is due to the C–O–C ether bonds of the repeating polyether structure and the absorbance near 1250 cm^−1^ is consistent with the aryl C–O stretch (Fig. 2a). These two main spectral features are distinguishable from the spectrum of the PBS buffer found in the second half of the diafiltration process (Fig. 2b). To estimate an approximate limit of detection of surfactant, the absorbance of the 1094 cm^−1^ C–O–C peak vs concentration was evaluated. Normally a linear relationship between absorbance and concentration allows an estimate of the concentration at 0.001 absorbance units (au). This, conservative, estimated limit of detection is approximately 0.058% or 0.58 g/L under the conditions the spectra were collected. This limit of detection was improved in subsequent work by increasing the number of scans collected and the application of multivariate models.

### 3.2 Laboratory study using manufacturing in-process samples with spiked surfactant

To begin the evaluation of mid-IR, a laboratory study was performed using a limited number of in-process samples spiked with surfactant using Nicolet iS50 FTIR spectrometer. In an effort to understand process variability and the impact of the PBS buffer, samples of the final diavolume exchange (exchange 15) were collected from two 50KDa TFF runs and known amounts of surfactant added, creating a series of synthetic standards spanning a concentration range of the surfactant (0 to 5%).

**Fig. 3:**
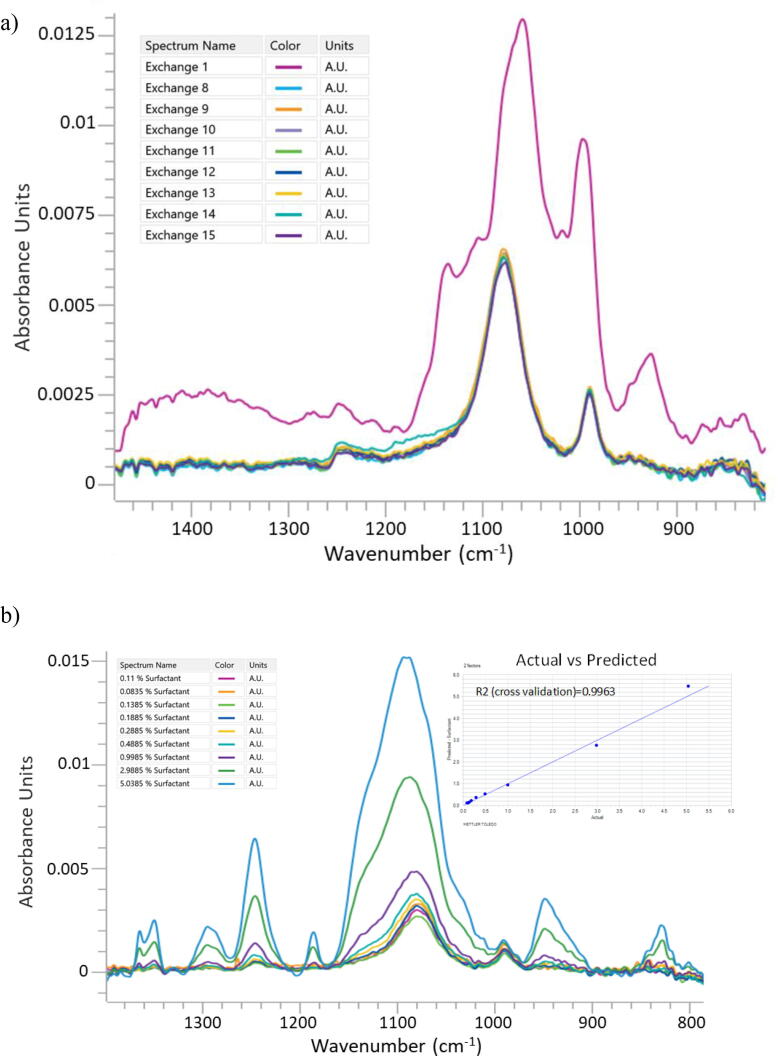
a) MIR Spectra of vaccine drug substance samples from a typical 50KDa TFF process; b) MIR spectra of exchange 15 vaccine drug substance sample (graph) spiked with increasing amounts of surfactant and PLS model actual vs predicted cross-validation plot (insert).

Fig. 3a shows spectra of process samples collected from a 50KDa TFF process. Note the spectrum of exchange volume 1 in Fig. 3a is dominated by the high concentration of sucrose found in the starting buffer. While surfactant concentration in exchanges 8 through 15 is decreasing, the major bands in the spectra are associated with the presence of 30 mM PBS buffer. The exchange 15 sample was then spiked with increasing amounts of surfactant to create a set of synthetic surfactant standards in a matrix of process material (Fig. 3b).

As shown in Fig. 3b, the spectral regions associated with the presence of the surfactant respond as expected with concentration, which was measured by the rapid HPLC method (Fig. S1). Using these spectra to create a PLS model enables an evaluation of the overlap of the PBS buffer peaks (Fig. 3a, insert). Combining the spectra, calculated surfactant concentrations over the range of 1300 cm^−1^ to 900 cm^−1^ resulted in the cross-validation plot (Fig. 3b inset). The clear correlation of surfactant concentration despite the spectral overlap of the PBS peaks indicated the feasibility of such an in-line measurement.

### 3.3 Feasibility of MIR for diafiltration step using process off-line samples

**Fig. 4:**
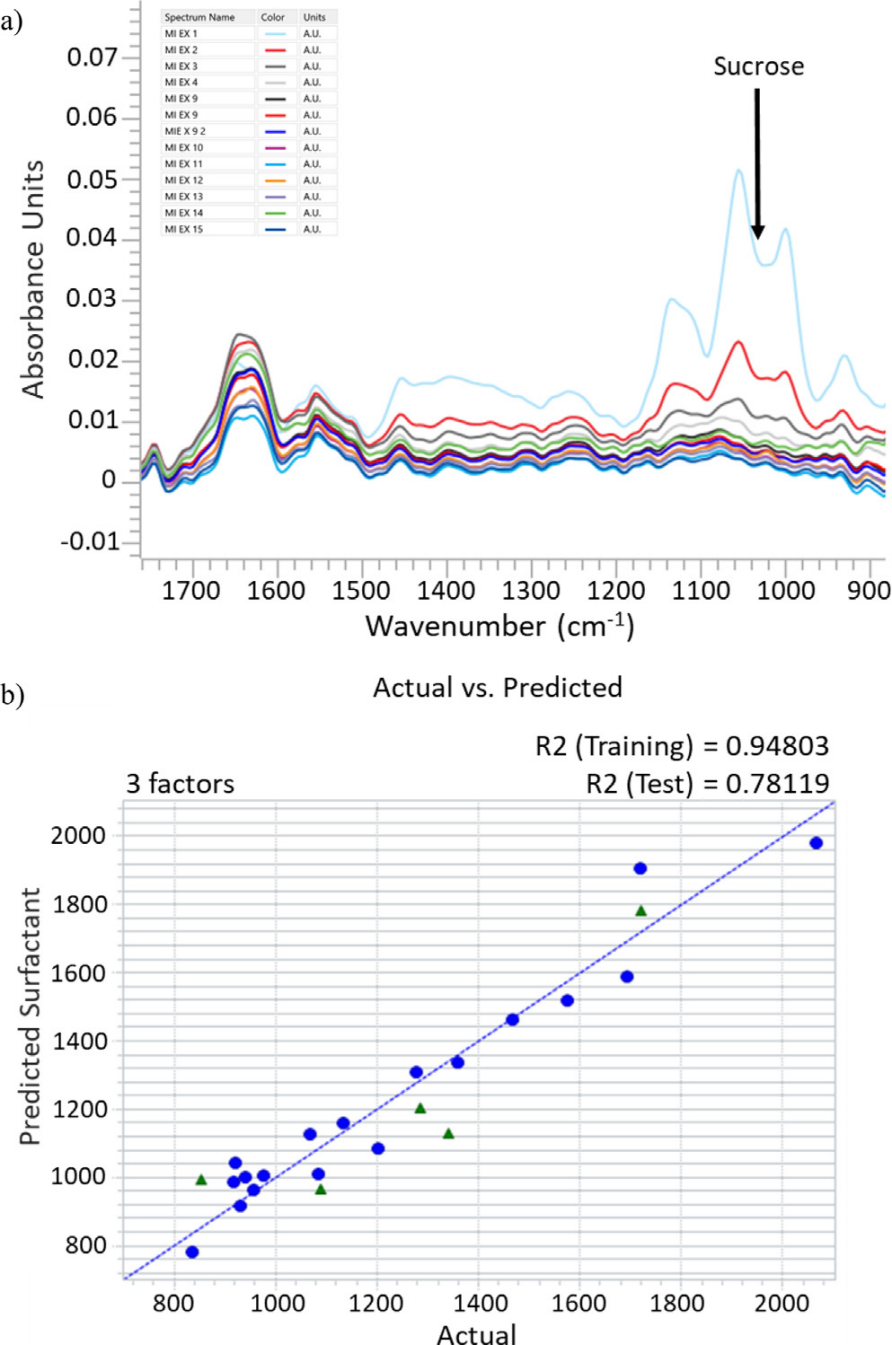
a) Process Off-line samples with PBS buffer subtracted. The scans are representative of four ultrafiltration runs. b) PLS model actual vs predicted plot with validation samples plotted in green triangles. Model includes 4 diafiltration runs (2 strain 2, 1 strain 1 and 1 strains 3). (For interpretation of the references to colour in this figure legend, the reader is referred to the web version of this article.)

The next step in the evaluation of an in-line infrared measurement was to work with true process samples in which the surfactant concentration was determined by the at-line rapid HPLC method and with an instrument similar to one that would be used for the process measurement. Despite the limited availability of process samples, a full set of samples were obtained from a 50KDa TFF run of viral strain 1 (Fig. 4a, Table S1). In addition, a subset of 4–10 diavolume exchanges from viral strains 2 and 3 (Table S1) were collected and analyzed. The overall trends for the decrease in surfactant concentration were the same between strains, however the rate of surfactant concentration change was higher for the 2 and 3 strains.

As in the lab study, only samples from exchange 8 on were used so as to not include the potential interference from the high concentrations of sucrose in exchanges between 1 and 7. Table S1 lists the samples and associated surfactant concentration as calculated by the at-line HPLC method. The range of surfactant concentrations is repeatable across the four TFF runs ranging from about 2000 µg/ml to between 800 and 950 ug/ml. The target surfactant concentration is calculated for each run, based upon the concentration of virus in each batch.

Fig. 4a, shows the spectra collected from the strain 1 sample set with the PBS buffer subtracted. Without the spectral contributions from the PBS buffer, features from the surfactant and soluble protein are more apparent. Note that the amide l and ll bands (approximately 1640 cm^−1^ and 1545 cm^−1^ respectively) from the soluble protein show subtle changes in intensity and shape throughout the samples. These changes are to be expected as the rate of diafiltration, thus total system volume, varies through the run.

In order to test feasibility of a surfactant measurement, the spectra of strain 1, strain 2, and strain 3 diafiltration exchanges (Table S1) were analyzed in conjunction with the HPLC derived surfactant concentrations and were used to create an exploratory PLS model (Fig. 4b). This simple model contains only 23 samples, uses a narrow spectral region (1284 cm^−1^ to 972 cm^−1^) and a two-point baseline across the same region. Five of the samples were reserved as validation samples. Using only 3 latent variables, the predictions of the test set spectra are shown in Fig. 4b and considering the small training set size, show promise. Further confirmation of this preliminary PLS model can be found in close examination of the latent variables. Fig. S2 shows the second latent variable used in the model and the similarity between the spectral features of the surfactant reference spectrum. Specifically, the aryl C–O stretch near 1245 cm^−1^ and the C–O–C ether stretch maxima just below 1100 cm^−1^ indicate the second latent variable largely models the presence of the surfactant.

### 3.4 Small scale TFF experiment with ReactIR probe to measure surfactant concentration in-line

The promising results of the process samples in the previous section were used to justify a small-scale TFF test that would use (rare and expensive) fresh process material and simulate an at scale process measurement. Goals of this work included: to confirm that in-line data will be similar to prior work with off-line samples, to identify any unknown aspects of a real-time measurement, and introduce the instrumentation to the manufacturing support group and gather feedback on adaptation of an in-line measurement in a manufacturing environment.

An aliquot of approximately 700 mL of viral drug substance material was used to run a TFF experiment with a ReactIR probe placed in the retentate vessel during the run. Reference spectra of the ending PBS buffer were also collected for use during data analysis.

As in the off-line sample investigations, data was collected after the sucrose was removed in the first 7 exchanges. Normally at process scale, exchanges require approximately 15 mins per diavolume however, in this experiment, each exchange required only approximately 3 min. As such, the process was placed in recirculation mode between each exchange volume so that 4 to 5 representative infrared spectra could be collected at each exchange. After the last infrared spectrum was collected, a sample was collected from the retentate vessel for off-line HPLC surfactant determination. At the conclusion of the TFF process, the process was placed in recirculation and infrared data collected for an additional two hours. Following the two-hour hold, small additions of surfactant were made to the retentate vessel and additional samples collected for at-line HPLC analysis. The resulting solution was then recirculated overnight, and a final HPLC sample collected in the morning.

**Fig. 5:**
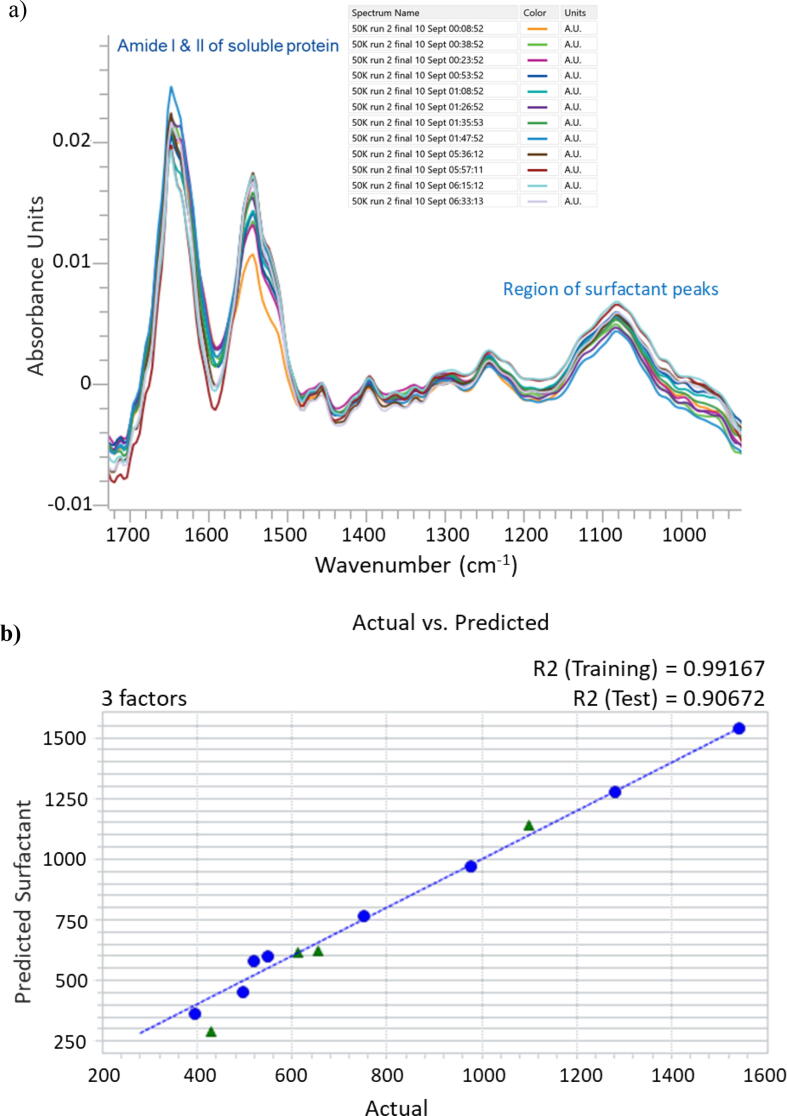
a) In-line MIR spectra of a small-scale diafiltration exchanges; b) Combining the MIR with HPLC - PLS model for the exchanges 8 to 15.

Fig. 5a, shows the infrared spectra associated with each off-line sample collection time during the TFF experiment, surfactant additions and overnight recirculation. A reference spectrum of the final PBS buffer was subtracted from each of the spectra shown in Fig. 5a to eliminate the interference from the PBS buffer peaks and make to visualization of the soluble protein drug substance and surfactant peaks possible.

Following the same approach used with the multi-strain off-line sample spectra, spectra from the 50KDa TFF experiment collected at the time a sample for off-line surfactant analysis were used to construct a PLS model. As before, the goal of this model was to test for consistency and correlation in the data set. The model was composed using 8 training set samples, 4 samples were reserved for a validation set, the region from 1300 cm^−1^ to 1000 cm^−1^ and 3 latent variables. Fig. 5b is a plot that shows cross validation data for the spectra used in the model and then the results of the training set data used to test the model, which is the actual vs predicted surfactant concentrations using the 3 latent variable PLS model. Superimposed in the green triangles are the predictions of the validation set samples.

**Fig. 6:**
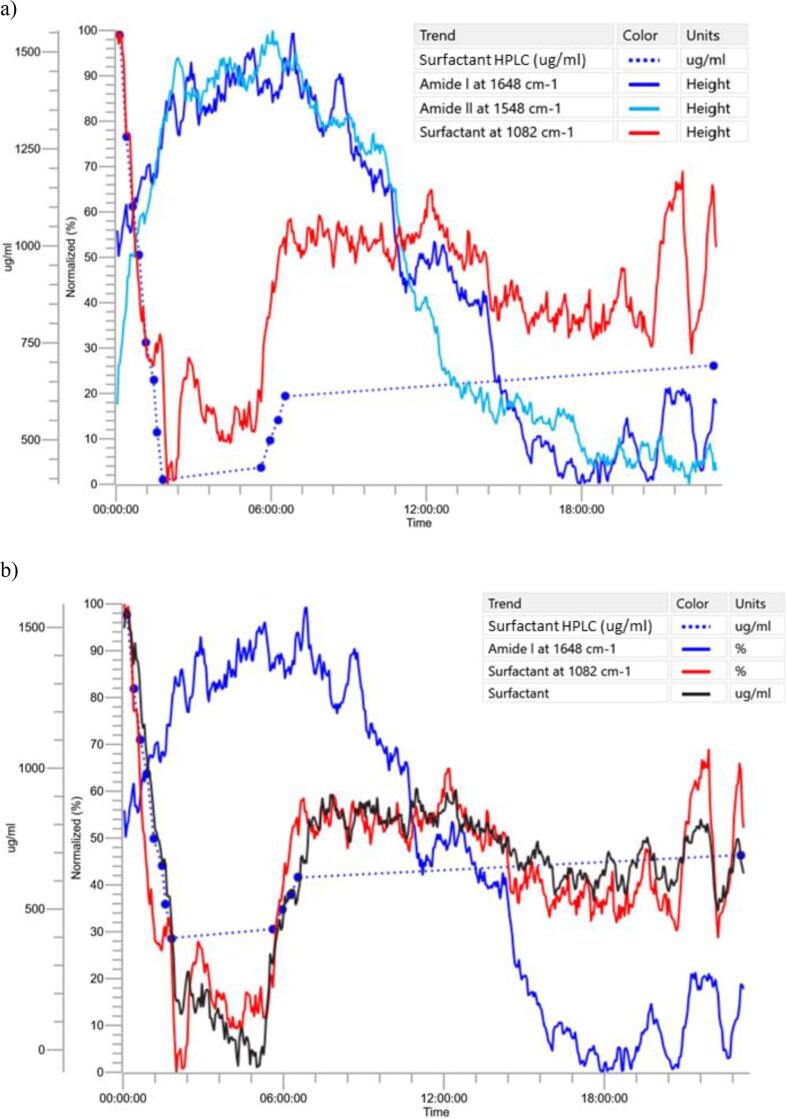
a) Peak intensity trends versus time for the surfactant and drug substance b) Overlay of peak intensity trends and surfactant PLS model predictions.

During the TFF experiment, the ReactIR generated an infrared spectrum every 3 min. Monitoring the infrared regions of interest included, the surfactant peaks and the amide carbonyl regions which resulted in trend plots of peak intensity vs time. Fig. 6a shows the infrared peak trend of the 1082 cm^−1^ peak associated with the surfactant. Note that the trend generally correlates well with the off-line surfactant concentrations measured by HPLC (Table 1) represented by the blue circles. Monitoring peak intensities is expedient and does not require calibration, however at low surfactant concentrations, this approach may not provide the accuracy and precision required for an in-line measurement.

**Table 1:** Surfactant concentration measured by HPLC and predicted by FTIR.

Surfactant concentration measured by HPLC, µg/ml	Surfactant concentration predicted by FTIR, µg/ml
1544	1535
1282	1275
1101	1140
978	965
752	762
654	618
519	580
397	357
429	287
499	449
550	594
612	615
692	582

The concentration of surfactant measured by HPLC were compared with those predicted by FTIR (Table 1).

Fig. 6b shows the model predictions for the experiment in black with the at-line HPLC data plotted in the blue dotted line. Despite the small dataset, prediction of surfactant concentration using the infrared model is surprisingly accurate and may reveal details of the interaction of surfactant and protein. The limited number of samples available to create the model used in this portion of the study, restricts interpretation of the results to merely; spectral changes can be correlated to off-line surfactant concentrations and the spectra are consistent within the experiment.

## 4 Discussion

There are many aspects to evaluating the suitability of a PAT measurement for routine commercial scale use. Early feasibility work normally consists of understanding and defining the measurement requirements such as: value of the measurement to the manufacturing or R&D organization, analyte(s) of interest, nominal concentration range(s) of analytes, matrix in which the measurement will be made, and how best to interface to the process. In the case study presented here, there were numerous constraints associated with working with a live virus process, such as the limited availability of process samples and the difficulty testing the measurement under conditions comparable to commercial scale.

Ultimately, the totality of the results of this limited study, were found sufficient to justify additional work so that this measurement could be applied to commercial scale process control. This real-time measurement of low concentrations of surfactant in a changing matrix of live virus and buffer is complex, and several risks were identified that can only be effectively addressed with further development and testing of this measurement. In this study, a small aliquots of drug substance were used. At manufacturing scale, the IR probe will be installed into a stainless-steel filtration tank and subjected to cleaning by sodium hydroxide. Once cleaning cycle complete, the product will be filled into the tank aseptically for the diafiltration to begin. Further, the drug substance is subjected to the sterile filtration, then diluted aseptically prior to filling of drug product.

Despite the relative ease of modeling using the limited data sets from both multi-strain process samples and the small scale TFF experiment, there are variables that will require further investigation. During a normal production year, three viral strains are produced for each geographical region. There are numerous geographical regions and therefore many viral strains are processed annually. Only further work will identify if a common surfactant model will provide sufficient accuracy for each strain. In addition, normal seasonal process variability both within a single strain and across multiple strains will require more at scale data to determine the impact of this critical variable.

Results from the small scale TFF study indicate the possibility of an equilibrium between the surfactant, drug substance, other soluble proteins and the TFF apparatus. Careful inspection of the infrared predicted surfactant trend in Fig. 6b, shows that after the last exchange sample point, the FTIR predicted surfactant concentration continued to decrease for approximately 30 min while the system was recirculating. Further experiments will be required to understand if this effect is an artifact of the experiment, related to the small scale TFF apparatus and whether this effect occurs at commercial scale. These observations would have been missed without the in-line measurement.

In the evaluation of PAT measurements, feasibility studies may be limited in scope by processing conditions, availability of representative samples and unknown relationships between small or pilot scale and commercial scale process variables. The existence of these unknowns may highlight risk associated with evaluating the performance of an in-line measurement. It may not be possible to mitigate these risks with carefully designed experiments compared with installing and gathering true process data at scale. In these situations, it is helpful to recognize the additional advantages to in-line measurements that were considered during this evaluation. The primary financial driver for an in-line measurement was found to be the costs associated with maintaining and staffing the off-line measurement whenever the 50KDa TFF process was run. These savings were estimated to be approximately $1.5MM per year. In addition to the financial return on investment associated with the reduction of off-line sampling required for each TFF run, data from an in-line measurement will provide an “overview” of the TFF process including the rate of sucrose clearance, the rate of exchange of buffers and the rate of surfactant reduction. These real-time trends could be used to detect abnormalities in the process associated with mechanical failures and membrane performance. In either case, the rapid detection of a process upset improves the chances of corrective action taken before the batch is at risk. Finally, real-time measurements may improve the understanding of the relationship between surfactant concentration, mixing time and protein structure.

## 5 Conclusion

The results of these three studies demonstrate that an in-line infrared measurement combined with a multivariate model can predict surfactant concentrations in the range encountered during the TFF process step. In view of the constraints on the availability of representative process samples and process material, the data presented herein demonstrate the feasibility of a real-time surfactant measurement. Based on the results of the off-line and in-line studies ReactIR is recommended as a PAT solution to measure concentration of surfactant in-line at manufacturing scale.

## CRediT authorship contribution statement

**Jessie Payne:** Study and publication lead in Analytical Sciences. **James Cronin:** Data generation and interpretation. **Manjit Haer:** Data generation and interpretation. **Jason Krouse:** Data generation and interpretation. **Bill Prosperi:** Data generation and interpretation. **Katherine Drolet-Vives:** Data interpretation. **Matthew Lieve:** Data generation and interpretation. **Mike Soika:** Data generation and interpretation. **Matthew Balmer:** Study and publication director in Analytical Sciences. **Marina Kirkitadze:** Study and publication director in Analytical Sciences.

## Declaration of Competing Interest

The authors declare that they have no known competing financial interests or personal relationships that could have appeared to influence the work reported in this paper.
