# Supplementary material for: In-line monitoring of surfactant clearance in viral vaccine downstream processing
Source: Comput Struct Biotechnol J. 2021 Mar 26;19:1829–37. doi: 10.1016/j.csbj.2021.03.030 (PMC8056174; doi:10.1016/j.csbj.2021.03.030)
Supplement: Supplementary data 1 [file mmc1.docx]

**Supplementary information**


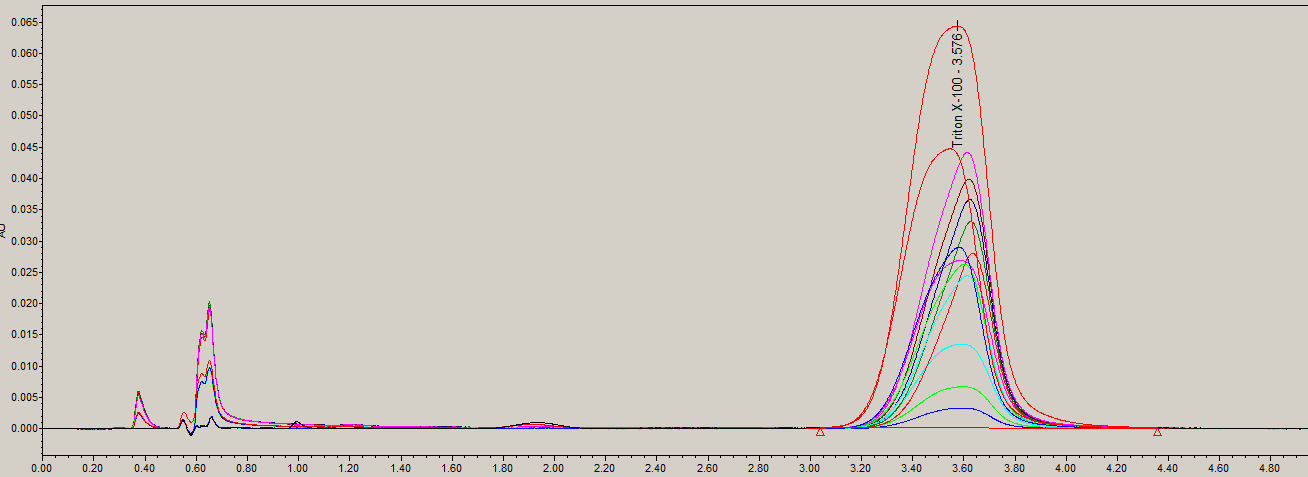

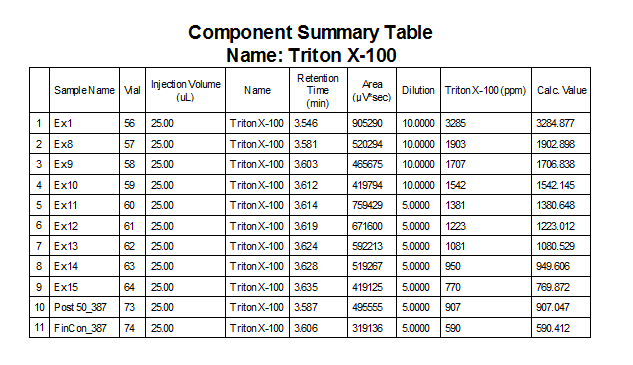


Component Summary Table Name: Surfactant

**Surfactant (ppm)**

**Surfactant**

**Surfactant**

**Surfactant**

**Surfactant**

**Surfactant**

**Surfactant**

**Surfactant**

**Surfactant**

**Surfactant**

**Surfactant**

**Surfactant**

Surfactant

**Figure S1.** Surfactant concentration measured by HPLC for the lab-based off-line study.

**Table S1.** Table 1: Surfactant concentrations measured by HPLC in off-line in-process samples

Strain 3 Strain 3 Strain 1 Strain 2

**Figure S2**: Second latent variable shows similarities to the surfactant spectrum in the range 1284 cm^-1^ to 972 cm^-1^
